# Responses to donor proliferation in Ghana’s health sector: a qualitative case study

**DOI:** 10.2471/BLT.14.141614

**Published:** 2014-10-27

**Authors:** Sarah Wood Pallas, Justice Nonvignon, Moses Aikins, Jennifer Prah Ruger

**Affiliations:** a Yale School of Public Health, PO Box 208034, New Haven, CT 06520-8034, United States of America (USA).; b School of Public Health, University of Ghana, Accra, Ghana.; c Perelman School of Medicine, University of Pennsylvania, Philadelphia, USA.

## Abstract

**Objective:**

To investigate how donors and government agencies responded to a proliferation of donors providing aid to Ghana’s health sector between 1995 and 2012.

**Methods:**

We interviewed 39 key informants from donor agencies, central government and nongovernmental organizations in Accra. These respondents were purposively selected to provide local and international views from the three types of institutions. Data collected from the respondents were compared with relevant documentary materials – e.g. reports and media articles – collected during interviews and through online research.

**Findings:**

Ghana’s response to donor proliferation included creation of a sector-wide approach, a shift to sector budget support, the institutionalization of a Health Sector Working Group and anticipation of donor withdrawal following the country’s change from low-income to lower-middle income status. Key themes included the importance of leadership and political support, the internalization of norms for harmonization, alignment and ownership, tension between the different methods used to improve aid effectiveness, and a shift to a unidirectional accountability paradigm for health-sector performance.

**Conclusion:**

In 1995–2012, the country’s central government and donors responded to donor proliferation in health-sector aid by promoting harmonization and alignment. This response was motivated by Ghana’s need for foreign aid, constraints on the capacity of governmental human resources and inefficiencies created by donor proliferation. Although this decreased the government’s transaction costs, it also increased the donors’ coordination costs and reduced the government’s negotiation options. Harmonization and alignment measures may have prompted donors to return to stand-alone projects to increase accountability and identification with beneficial impacts of projects.

## Résumé

**Objectif:**

Étudier la manière dont les organismes donateurs et les agences gouvernementales ont répondu à la prolifération des donateurs qui ont apporté un soutien au secteur de la santé du Ghana entre 1995 et 2012.

**Méthodes:**

Nous avons interrogé 39 informateurs clés issus des organismes donateurs, du gouvernement central et des organisations non gouvernementales de la ville d’Accra. Ces personnes interrogées ont été expressément sélectionnées pour fournir les visions locales et internationales des trois types d'institution. Les données recueillies auprès des personnes interrogées ont été comparées avec des documents pertinents, comme des rapports et des articles médiatiques, collectés au cours des entrevues et par le biais de recherches en ligne.

**Résultats:**

La réponse du Ghana à la prolifération des donateurs a inclus la création d'une approche à l'échelle du secteur, un transfert vers le soutien du budget du secteur, l'institutionnalisation du Groupe de Travail du Secteur de la Santé et l'anticipation du retrait des donneurs après le passage de pays à revenu faible à pays à revenu moyen inférieur. Les principaux thèmes ont inclus l'importance du leadership et du soutien politique, l'internalisation des normes pour l'harmonisation, l'alignement et la propriété, la tension entre les différentes méthodes utilisées pour améliorer l'efficacité de l'aide et un transfert vers un paradigme de responsabilité unidirectionnelle pour la performance du secteur de la santé.

**Conclusion:**

De 1995 à 2012, le gouvernement central du pays et les donateurs ont répondu à la prolifération des donateurs dans l'aide apportée au secteur de la santé en favorisant l'harmonisation et l'alignement. Cette réponse était motivée par le besoin du Ghana en aides étrangères, les limitations en matière de capacité des ressources humaines gouvernementales et le manque d'efficacité créé par la prolifération des donateurs. Bien que cela ait diminué les coûts de transaction du gouvernement, cela a également augmenté les coûts de coordination des donateurs et réduit les options de négociation du gouvernement. Les mesures d'harmonisation et d'alignement peuvent avoir causé le retour des donateurs à des projets autonomes visant à augmenter la responsabilité et l'identification, avec des effets bénéfiques pour les projets.

## Resumen

**Objetivo:**

Investigar el modo en que los donantes y las agencias gubernamentales respondieron a la proliferación de donantes que prestan apoyo al sector sanitario de Ghana entre 1995 y 2012.

**Métodos:**

Entrevistamos a 39 informantes clave de organismos donantes, el gobierno central y organizaciones no gubernamentales en Accra. Se seleccionó deliberadamente a los encuestados para ofrecer puntos de vista locales e internacionales de los tres tipos de instituciones. Se compararon los datos recogidos de los encuestados con el material documental pertinente, como informes y artículos de prensa, recopilado durante las entrevistas y a través de la investigación en línea.

**Resultados:**

La respuesta de Ghana a la proliferación de donantes incluyó la creación de un enfoque sectorial, un cambio hacia el apoyo presupuestario sectorial, la institucionalización de un Grupo de Trabajo del Sector de la Salud y la anticipación de la retirada de los donantes tras el cambio del país de ingresos bajos a ingresos medios bajos. Los temas clave incluyeron la importancia del liderazgo y el apoyo político, la interiorización de las normas para la armonización, la estandarización y la propiedad, la tensión entre los distintos métodos utilizados para mejorar la eficacia de la ayuda, así como un cambio hacia un paradigma de rendición de cuentas unidireccional para el rendimiento del sector sanitario.

**Conclusión:**

Entre 1995 y 2012, el gobierno central y los donantes del país respondieron a la proliferación de donantes para ayudar al sector sanitario mediante la promoción de la armonización y la estandarización. Esta respuesta vino motivada por la necesidad de ayuda internacional de Ghana, las limitaciones de la capacidad de los recursos humanos gubernamentales y la ineficiencia creada por la proliferación de donantes. Pese a que esta situación disminuyó los costes de transacción del gobierno, también aumentó los costes de coordinación de donantes y redujo las opciones de negociación del gobierno. Las medidas de armonización y estandarización pueden haber incitado a los donantes a volver a proyectos independientes para aumentar la rendición de cuentas y la identificación con los efectos beneficiosos de los proyectos.

## ملخص

**الغرض:**

تحري كيفية استجابة الجهات المانحة والأجهزة الحكومية لانتشار الجهات المانحة التي تقدم المعونة إلى قطاع الصحة في غانا في الفترة من عام 1995 إلى عام 2012.

**الطريقة:**

أجرينا مقابلات مع 39 من مبلغي المعلومات الرئيسيين من الوكالات المانحة والحكومة المركزية والمنظمات غير الحكومية في أكرا. وتم اختيار هؤلاء المستجيبين على نحو مقصود لتقديم وجهات النظر المحلية والدولية من أنواع المؤسسات الثلاث. وتم مقارنة البيانات التي تم جمعها من المستجيبين بالمواد الوثائقية ذات الصلة - أي التقارير والمقالات الإعلامية - التي تم جمعها خلال المقابلات وعن طريق الأبحاث على شبكة الإنترنت.

**النتائج:**

اشتملت استجابة غانا لانتشار الجهات المانحة على إنشاء نهج على نطاق القطاع، والتحول إلى دعم ميزانية القطاع، والتنظيم المؤسسي للفريق العامل في قطاع الصحة وتوقع انسحاب الجهات المانحة عقب تغير البلد من بلدان الدخل المنخفض إلى بلدان الدخل المنخفض - المتوسط. وتضمنت الموضوعات الرئيسية أهمية القيادة والدعم السياسي وإضفاء الصبغة الداخلية على معايير التجانس والمواءمة والملكية والتوتر بين مختلف الأساليب المستخدمة لتحسين فعالية المعونة والتحول إلى نموذج أحادي الاتجاه للمساءلة من أجل أداء قطاع الصحة.

**الاستنتاج:**

في الفترة من عام 1995 إلى 2012، استجابت الحكومة المركزية بالبلد والجهات المانحة إلى انتشار الجهات المانحة في معونة قطاع الصحة عن طريق تعزيز التجانس والمواءمة. وكان الدافع وراء هذه الاستجابة حاجة غانا للمعونة الخارجية والقيود على قدرة الموارد البشرية الحكومية وأوجه عدم الكفاءة الناشئة عن انتشار الجهات المانحة. وعلى الرغم من أن هذا الإجراء أدى إلى خفض تكاليف المعاملات الحكومية، إلا أنه أدى كذلك إلى زيادة تكاليف التنسيق للجهات المانحة وإلى خفض خيارات التفاوض الحكومية. ومن المحتمل أن تطالب تدابير التجانس والمواءمة الجهات المانحة بالعودة إلى المشاريع القائمة بذاتها لزيادة المساءلة وتحديد الآثار المفيدة للمشاريع.

## 摘要

**目的:**

调查在1995年和2012年之间捐助者和政府机构如何回应为加纳卫生部门提供援助的捐助者扩增。

**方法:**

我们采访了阿克拉捐助机构、中央政府和非政府组织的39位关键信息提供者。这些受访者经过特意挑选以提供三种类型机构的本地和国际观点。将从受访者处收集的数据与通过访谈和在线研究收集的相关文献材料（例如报告和媒体文章）进行比较。

**结果:**

加纳对捐助者增加的回应包括创建全部门方法、转向部门预算支持、卫生部门工作组制度化，以及国家从低收入转变为中下游收入水平之后捐助者退出的预期。关键主题包括领导和政治支持的重要性、协调规范的内化、统调和所有权、用来提高援助效果的不同方法之间的矛盾，以及为提高卫生部门绩效而转向单向责任范式。

**结论:**

在1995至2012年，该国中央政府和捐助者通过促进协调和统调对卫生部门捐助者的增加做出回应。这种回应加纳对外国援助的需要、政府人力资源能力的局限以及捐助者增加造成的低效率促成了这种回应。虽然这降低了政府的事务成本，但也增加了捐助者的协调成本并减少政府的协商选项。协调和统调措施可能促使捐助者回到独立项目，以增加责任承担以及对这些项目有益影响的认同。

## Резюме

**Цель:**

Исследовать, как доноры и правительственные учреждения отреагировали на рост количества доноров, предоставлявших помощь сектору здравоохранения Ганы в 1995-2012 гг.

**Методы:**

Были опрошены 39 ключевых источников информации из донорских учреждений, центральных правительственных и неправительственных организаций в Аккре. Эти респонденты были целенаправленно выбраны для определения местной и международной точек зрения из трех типов учреждений. Данные, собранные у респондентов, были сопоставлены с соответствующими документальными материалами, например, отчетами и статьями в средствах массовой информации, собранными во время интервью и в ходе онлайн-исследований.

**Результаты:**

Реакция Ганы на увеличение числа доноров включала в себя создание секторального подхода, переход к бюджетной поддержке сектора, институционализацию Рабочей группы сектора здравоохранения и предвидение ухода доноров в связи с превращением Ганы из страны с низким уровнем доходов в страну с уровнем доходов ниже среднего. К числу ключевых тем относились следующие: значение руководства и политической поддержки, интернализация норм по гармонизации, согласованию и владению, противоречия между различными методами, используемыми для повышения эффективности помощи, и переход к однонаправленной парадигме подотчетности для оценки эффективности сектора здравоохранения.

**Вывод:**

В 1995-2012 гг. центральное правительство и доноры страны отреагировали на увеличение количества доноров в сфере помощи сектору здравоохранения путем содействия гармонизации и согласованию. Подобная реакция мотивировалась потребностью Ганы в иностранной помощи, ограниченными возможностями правительственных кадровых ресурсов и нерациональностью, возникшей в результате увеличения числа доноров. Хотя, с одной стороны, это привело к снижению операционных издержек правительства, с другой стороны, выросли расходы на координацию действий доноров и сократился выбор правительства при проведении переговоров. Меры по гармонизации и согласованию могли бы побудить доноров вернуться к реализации самоокупаемых проектов для повышения подотчетности и идентификации с положительным воздействием проектов.

## Introduction

Since the 1990s, development aid for health and the number of donor organizations providing such aid have grown dramatically.^1^^–^^3^ It has been suggested that, as the number of donors increases, the transaction costs of aid-recipient countries increase, the performance incentives for donors and recipients diminish, the quantity and quality of human resources in the recipient government bureaucracy decrease and corruption within recipient countries increases.^4^^–^^10^ Between 2002 and 2012, such potential negative impacts provided motivation for international policy agreements on aid effectiveness^11^^–^^14^ – notably the 2005 Paris Declaration on Aid Effectiveness and the 2008 Accra Agenda for Action.^13^ The agreements promoted donor harmonization, donor alignment with recipient-country systems, country ownership, managing for results, and mutual accountability – i.e. the so-called aid effectiveness principles.^6^^,^^13^ Although there were earlier attempts to improve donor coordination – e.g. by the promotion of sector-wide approaches^15^^–^^17^ – donor proliferation and consensus around the current policy response have recently changed the landscape of development aid for health. It has become increasingly important to understand how recipient countries are responding to donor proliferation in health-sector aid. Unfortunately, there are few cross-country quantitative data on this topic. Data on the implementation of the 2005 Paris Declaration on Aid Effectiveness are collected by the Organisation for Economic Co-operation and Development^18^ but do not capture the full range of aid-effectiveness activities, are not disaggregated by sector and do not permit assessment of why certain aid effectiveness principles are adopted by some countries but resisted by others. It has been suggested that donors and recipients may resist aid coordination because it may weaken the recipient government’s negotiating position,^4^^,^^6^^,^^7^ increase aid volatility^19^ or impose new costs.^6^^,^^10^^,^^20^

**Fig. 1:**
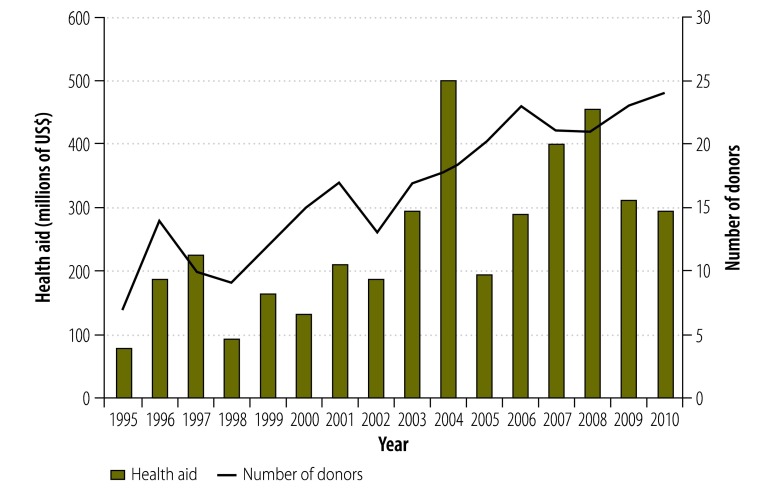
Number of donors and volume of health aid received by Ghana, 1995–2010

This article presents findings from a qualitative case study of the responses to a proliferation of donor aid to the health sector in Ghana. Between 1995 and 2010, Ghana gained 17 such donors (Fig. 1) – more than most other countries that received health-sector aid during this period.^21^ In adopting policies for donor coordination in its health sector during the 1990s^22^ and establishing multi-donor budget support and an associated policy dialogue mechanism in 2003,^23^^,^^24^ Ghana established many of the practices that were subsequently recommended during international fora to improve aid effectiveness.^11^^–^^13^ Since the 1990s, Ghana has transitioned from an indebted low-income country to a rapidly growing lower-middle income economy^23^^–^^25^ and has experienced improvements in multiple health indicators (Table 1).^25^^,^^26^ Analysing the case of Ghana may offer an early indication of how international policies to improve aid effectiveness may unfold in other low- or middle-income countries.

US$: United States dollars.

Notes: The count is the sum of the net number of donors that reported providing any official development assistance for sector codes 120, for general and basic health, 130, for population and reproductive health, or 140, for water and sanitation, in the Organisation for Economic Co-operation and Development’s (OECD’s) Creditor Reporting System. Donors that provided aid to multiple sector codes were only counted once. This count underestimates Ghana’s true magnitude of donor proliferation as it does not include donors that do not report to the OECD – e.g. Brazil, China, India and the Russian Federation – or private corporations and foundations other than the Bill & Melinda Gates Foundation. Furthermore, OECD member states are counted as single donors even though, in a single member state, there may be multiple government agencies disbursing aid. Volumes of aid are shown in United States dollars, as valued in 2009.

Data source: OECD.^21^

**Table 1:** Selected health indicators, Ghana, 1995 and 2009

Health indicator	Year	
	1995	2009
**Mortality rates**		
Infant (deaths before 1 year-of-age per 1000 live births)	70.6	51.3
Adult female (deaths between the ages of 15 and 60 years per 1000 women)	267.4^a^	230.3
Adult male (deaths between the ages of 15 and 60 years per 1000 men)	304.3^a^	259.8
Maternal (deaths per 100 000 live births)	540	350^b^
**DTP3 immunization coverage among 1-year-olds (%)**	70	94
**Tuberculosis case detection rate (newly notified cases as percentage of estimated number of incident cases)**	30	68
**Use of improved drinking water sources (% of population)**	63	82

DTP3: third round of diphtheria, tetanus toxoid and pertussis immunization.

^a^ Value for 1997.

^b^ Value for 2008.

Data sources: World Bank^25^ and World Health Organization.^26^

## Methods

We chose to use a retrospective qualitative case study because such studies permit analysis of complex multi-causal phenomena within real-world settings.^27^^,^^28^ We investigated Ghana’s experience of donor proliferation in health-sector aid between 1995 and 2012 – i.e. over a period that included the years before and after major global growth in health-sector aid and the years in which several international agreements on improving aid effectiveness were made.^1^^–^^3^^,^^11^^–^^14^ In Accra, we interviewed key informants who were individuals with current or previous work experience overseeing or implementing health-sector aid in Ghana in (i) central government agencies, (ii) donor organizations and (iii) nongovernmental organizations. We used purposive sampling to ensure inclusion of local and international views from all of these three types of organizations. The key informants were identified either via online searches – of the websites of organizations that, according to the Organisation for Economic Co-operation and Development,^21^ had provided or received health-sector aid in Ghana between 1995 and 2012 – or via snowball sampling.^28^

Interviews were requested by telephone and email using a standard script. The interviews were conducted in Accra during September–November 2012 in a location of the respondent’s choosing – typically the respondent’s office – with only the interviewer and one or two respondents present. One interview was conducted by telephone in February 2013. The interviewer used an interview guide that listed standard initial questions and optional follow-up questions. She took notes during each interview and – if the interviewees agreed – made a digital audio recording of all of the questions and answers. No repeat interviews were conducted and transcripts were not returned to respondents for comment or correction. Respondents were recruited until theoretical saturation was achieved.^28^^,^^29^

Although 43 interviews were requested, only 35 – involving 39 key informants – were conducted. Eight informants were unable to be interviewed because the authors received no responses after repeated interview requests or because the informants claimed to be too busy. The key informants interviewed had managed health-sector aid in the government (*n* = 14), donors (*n* = 14) or civil society (*n* = 7). Most (69%) of the interviewees were Ghanaian.

The digital recordings and interview notes were transcribed and coded for key themes by one of the authors using the constant comparative method.^27^ The preliminary start list of codes – which was generated from the research questions and literature reviewed for the study – included donor entry, donor exit, aid increases, aid decreases, donor distribution, donor competition, recipient competition, recipient government control, donor control, donor coordination, recipient government prioritization of health, aid effectiveness, accountability and aid-package features. Additional codes that emerged from the transcripts were added to the coding tree, which included each code plus nested subcodes for examples, causes and consequences. Word (Microsoft, Redmond, USA) and ATLAS.ti version 7 (ATLAS.ti Scientific Software Development, Berlin, Germany) were used to manage the data and facilitate quotation retrieval. Data collected from the respondents were compared with the relevant documentary materials – e.g. reports and media articles – collected during interviews and through online research. A timeline of events and key themes was derived from the coded data and documentary materials.

### Ethical approval

The study protocol was reviewed and approved by the Yale Human Subjects Committee – as protocol 1207010568, with exemption from further review granted under 45 CFR 46.101(b)(2) – and the Ghana Health Service Ethical Review Committee – as protocol 03/09/2012. All respondents provided verbal or written informed consent before being interviewed.

## Results

The study identified a timeline of key events (Box 1) and themes that defined the trajectory of the central government’s and donors’ responses to donor proliferation in Ghana’s health sector and the factors that influenced these responses.

Box 1 **Timeline of key events in Ghana’s response to donor proliferation in health-sector aid**

- **Late 1980s–early 1990s**: donors begin to proliferate in Ghana’s health sector.^22^
- **Early 1990s–mid 1990s**: to address parallel donor systems and increased aid transaction costs, Ghana’s Ministry of Health develops a health-sector-wide approach, with a pooled funding account and common management arrangement for donors.^22^
- **1997**: Denmark, the United Kingdom of Great Britain and Northern Ireland and the World Bank – all early supporters of the health-sector-wide approach – become the first donors to commit funds to the pooled funding account.^22^
- **1997–2001**: Ministry of Health First Five Year Programme of Work is implemented under the health-sector-wide approach.^30^ The approach is perceived as a successful model, reducing transaction costs for government and attracting donors to the health sector with its strategic approach, donor coordination forum, and transparent financial management.
- **2002–2006**: Ministry of Health Second Five Year Programme of Work is implemented under the health-sector-wide approach.^31^ Donors begin to move funds from the pooled funding account managed by the Ministry of Health to the sector budget support mechanism managed by the Ministry of Finance and Economic Planning.^32^ This was motivated in part by a global trend towards budget support following the international agreements on aid effectiveness in this period. Channelling donor funds through the Ministry of Finance and Economic Planning causes delays in disbursement to the Ministry of Health and subnational health units.
- **2003**: Ghana Poverty Reduction Strategy is agreed between donors and the Ghanaian government. Donors begin providing general budget support to the government under the multi-donor budget support and policy dialogue mechanism.^24^
- **2005–2006**: Ministry of Health allows donors who have not participated in the health-sector-wide approach to sign a health-sector strategy agreement, to enable these donors to participate in the sector-wide dialogue platform.
- **2007–2011**: Ministry of Health Third Five Year Programme of Work is implemented, no longer under the health-sector-wide approach.^32^ A Health Sector Working Group for donor–government coordination is fully institutionalized. Donors meet regularly among themselves before each working group session, to agree on a common platform for engagement with the government – representatives from donor agencies are selected to serve, on a rotating basis, to liaise with the Ministry of Health. These donor coordination arrangements reportedly increase the time required for donors to manage their health-sector-aid portfolios.
- **2008**: Ghana hosts the Third High Level Forum on Aid Effectiveness, which produces the Accra Agenda for Action and brings renewed attention to the principles of harmonization, alignment and country ownership.
- **2009–2012**: new donors such as Israel and the Republic of Korea begin providing health-sector aid and participating in Health Sector Working Group meetings.
- **2010**: Ghana rebases its calculations of its gross domestic product and attains the status of a lower-middle income country.^25^
- **2012**: some traditional donors – e.g. Denmark, the Netherlands and the United Kingdom – indicate their intention to withdraw from sector budget support, to return to a more project-based approach and wind down development-aid operations in Ghana.

### Key themes in the responses

#### Leadership and political support

Key informants described how creation of a health-sector-wide approach was enabled by a cohort of catalytic leaders within the Ministry of Health and in-country donor champions who secured support from their headquarters and peers. For example, a respondent from the central government stated:

“When we were negotiating with the World Bank, it [the sector-wide approach] was not something that they would support at the time. But DfID [the United Kingdom’s *Department for International Development]*stood in the Ministry [of Health], supported the ministry, and when we were going for the negotiations with the World Bank … DfID went with the ministry team just to provide the necessary support to get the World Bank to come on board.”

The risk-taking required for the sector-wide approach was facilitated by the political cover extended to the Ministry of Health representatives who negotiated with donors. Another respondent from the central government stated:

“We had political support and confidence from the Minister [of Health] … ‘You take the risk, I’ll take the blame’ is what my boss said to me. We considered aid and we could say no; if donors complained … we had support from the Minister.”

When the cadre of sector-wide-approach pioneers left the Ministry of Health for international organizations, they were replaced by officials who had excellent technical skills but were perceived to lack the same leadership qualities and high-level political support as the previous generation.

#### Internalization of norms

Respondents suggested that, in attempts to improve the effectiveness of aid, the internalization of norms for harmonization, alignment and ownership resulted primarily from Ghana’s local history of aid coordination. However, from 2002 onwards, such internalization was reinforced by the international agreements on aid effectiveness. These agreements provided a common global rhetoric that could be used to describe local practices. Respondents reported that it was the early leadership, institutionalization and success of the health-sector-wide approach in Ghana that established harmonization, alignment and ownership as the normative standards for health aid to Ghana, well before such principles were codified in the 2005 Paris Declaration. As agreements on aid effectiveness were made at the international level between 2002 and 2008, donors and government in Ghana were developing new structures such as sector budget support, the Health Sector Working Group, and development-partner coordination pre-meetings before the working group’s sessions. These structures reflected a continuation of the norms established in Ghana under the sector-wide approach and an intensification of efforts to improve aid effectiveness in Ghana, as donors and government leveraged the new vocabulary and political momentum from international agreements to motivate and justify local action. Respondents who had previously worked in other countries or sectors were impressed by the strength of aid coordination in Ghana’s health sector. One respondent from a donor stated that:

“The level of coordination and commitment to coordination is very high here, not just talking but actually working a lot for the Ministry [of Health] and development partners.”

Respondents noted how bilateral donors that traditionally supported stand-alone projects in other countries had sought ways to use more aligned approaches in Ghana. Another respondent from a donor stated:

“USAID [the United States Agency for International Development], who in the past has had problems working through government institutions, are getting quite positive tendencies in that direction [in Ghana] … I think it [Ghana] is the only place that Japan gives sector budget support.”

The international agreements were a rhetorical touchstone for donors and government officials, even when not fully operationalized in practice. One respondent from the central government stated:

“[The donors say] ‘we consulted government’ but … everybody is still sending their individual consultants. The [donors’] global guidelines are disconnected from country-level aid effectiveness.”

#### Tension between aid effectiveness principles

Tensions emerged as the aid effectiveness principles of ownership, alignment, harmonization, managing for results, and mutual accountability were more intensively applied in Ghana’s health sector after the sector-wide approach. One tension reported in interviews was between donor harmonization and country ownership. Respondents suggested that the pre-meetings among donors strengthened the donors’ voice and bargaining power in discussions with the Ministry of Health while limiting the ministry’s ability to negotiate with individual donors. Comments by a respondent from civil society included:

“Donors are a club; they don’t undermine each other. So government cannot be tough with one donor and then go to another; the other donor will refuse.”

Similarly, a respondent from a donor stated:

“But they [the government] can’t shop around because we have this development-partner group, so we will tell each other if the government approaches [any of] us.”

Some donors, such as the Government of China, reportedly did not participate in pre-meetings, an arrangement that some of our respondents speculated was preferred by Ghana’s central government to access aid from sources outside the donor coordination group.

A second tension reported by some respondents was that between alignment and managing for results. While the Ghanaian Ministry of Health favoured donors to be aligned through the sector-wide approach, the shift to sector budget support – an even more aligned mechanism using the Ministry of Finance and Economic Planning’s normal fiscal channels – had introduced delays in disbursement to the Ministry of Health. A governmental respondent stated:

*“*Now we have to go to them [the Ministry of Finance and Economic Planning] to chase them for donor funding and also for Government of Ghana [funding].”

On the same topic, a respondent from a donor stated:

“The exit of donors from sector budget support has improved coordination because more time is spent on programmatic issues and less on where is the money from the MOFEP [Ministry of Finance and Economic Planning].”

Similar tensions were reported between the central government and disease-specific donors, such as donors who focused on the control and treatment of human immunodeficiency virus (HIV). Respondents from other donors saw such disease-specific donors as problematic because funds from disease-specific donors may not always be tailored to the Ghanaian government’s health priorities. However, several governmental respondents mentioned that – since they often provided large sums to support well defined activities – the disease-specific donors were often easier to manage than the donors that provided more diverse forms of aid.

Respondents also noted how – although each donor might like to be able to attribute a benefit in Ghana to the aid that the donor had itself provided – the sector-wide approach and sector budget support implemented in Ghana prevented such attribution of benefit to a single donor. Some respondents suggested that some donors were going back to supporting stand-alone projects. One respondent from a donor stated:

“We have a conservative government now that is focused on getting credit. There is visibility pressure. They see other donors doing this, claiming results, using the flag.”

#### Unidirectional accountability paradigm

Respondents described the sector-wide approach as being characterized by a sense of joint accountability between donors and the government, with the government taking the lead. However, some respondents reportedly found current donor–government relations to be increasingly characterized by a unidirectional accountability in which donors held the Ministry of Health entirely accountable for all relevant outcomes. For example, a governmental respondent stated:

“Pooling [donor aid] also increases the risk for the Ministry of Health. If they don’t meet one out of the 10 indicators, then every donor in the pool reduces by 10%, so it exposes the sector to risks and fluctuations … The Ministry of Health has already spent the money trying to achieve the indicator, but if the Ministry of Health only gets to 98% of the indicator, they get no money from [the development] partners.”

Respondents mentioned several reasons for the shift in accountability paradigms, including Ghana’s economic and capacity development over time and political changes in the donors’ home countries. One respondent from a donor said:

“We’re talking about phasing out … within the next five years it’s going to be much more commercial, political collaboration, not so much development … We’ve been in the health sector for 20-plus years and we’re looking at a country that has achieved lower-middle income status … Sometimes they say ...‘But in the past you used to help us with this’ and [we have to say]…‘But you know things are progressing as well and now you do it yourself’.”

Respondents suggested that strengthening Ministry of Health capacity to develop a policy agenda might restore a more balanced approach to accountability. A respondent from a donor stated:

“The capacity of individuals on the government side needs strengthening. If they don’t have the capacity to demand accountability from development partners, I could see things sort of falling apart. If development partners feel that the Ministry of Health knows what it is doing … then it gives the Ministry of Health more control.”

#### Minor themes

Minor themes discussed in the interviews included (i) the relative impacts of the sector-wide approach and sector budget support on Ghana’s central and subnational government units; (ii) coordination modalities among the central government units; and (iii) the interface between a democratic political environment and technical civil service processes. These minor themes are not presented in detail here as they primarily concern dynamics within the Government of Ghana rather than donor–government relations in response to donor proliferation.

## Discussion

The accounts of our respondents provide support for earlier predictions of the probable effects of responses to donor proliferation. Harmonization, alignment and ownership had reportedly reduced transaction costs for Ghana’s Ministry of Health, although the transaction costs for the donors – who needed to spend more time on coordination and extracting results from aggregate Ministry of Health reports – had increased. Donor–government dialogue platforms had facilitated information sharing while the internalization of aid-effectiveness norms – initially from Ghana’s local efforts at aid coordination and later reinforced by international agreements – had mitigated donor competition. However, donor coordination had limited the Ministry of Health’s negotiation options and made aid more volatile at certain points.

Data collected from the respondents also revealed several novel findings. First, they explained why donors and government officials had adopted the aid effectiveness principles in response to donor proliferation in Ghana’s health sector. Donor proliferation created parallel administrative systems and increased transaction costs for the Ministry of Health and a public health service that had relatively low capacity. At the same time, however, the Ghanaian government was reluctant to refuse any aid because it found itself in a weak fiscal position as it emerged from high indebtedness.^23^^,^^24^ This combination – of a high need for development aid, relatively limited management capacity within government and perceived inefficiencies from donor proliferation – prompted the Ministry of Health to adopt a strategy of retaining donors within the health sector while channelling aid through more streamlined approaches such as the sector-wide approach and sector budget support.

Second, the data we collected highlight the conditions that facilitated the adoption and maintenance of aid effectiveness principles as a response to donor proliferation. In Ghana, risk-taking leadership by both the government and donors was important in improving the coordination of health-sector aid in the face of donor proliferation. The individuals who launched the sector-wide approach were facilitated by political cover from senior officials and were willing to conflict with existing practices in their organizations. The sector-wide approach established norms of donor and government behaviour in Ghana’s health sector. These norms were reinforced by the later international agreements on aid effectiveness and facilitated adoption of the rhetoric and policy consensus promulgated in these agreements. Commitment to aid effectiveness principles may also have been facilitated by the Ghanaian government’s broader institutionalization of platforms for donor coordination – e.g. by the initiation of multi-donor budget support and the routine integration of aid into fiscal planning. A local history of aid coordination with strong government leadership may be an important condition for effective implementation of global agreements on aid effectiveness.

Third, the information from our respondents revealed a potential paradox in the application of aid effectiveness principles: as these principles are more completely applied, donors are less able to satisfy their internal institutional needs for attribution and accountability. At some point, a donor may choose to exit from sector-wide coordination efforts or pooled funding mechanisms so that it can reassert a donor-specific identity and increase its visibility by supporting stand-alone projects. The 2008–2009 global financial crisis and Ghana’s achievement of lower-middle income status in 2010 increased the probability that donors would change the ways in which they provided aid to Ghana. It appears that donors’ own institutional or political needs can override commitments to channel aid in ways that should maximize the health benefits.

Our study findings are subject to several limitations. Some key informants who were invited to participate in our study did not participate. Although theoretical saturation was achieved, it is not possible to know what insights such respondents might have contributed. Moreover, the informants’ responses may have been subject to social desirability or recall biases. Interviews were only conducted in Accra. If the views of informants working at subnational levels or in donors’ headquarters differ systematically from those of informants in central government or donor in-country offices, then our study may not have captured all views on donor proliferation in Ghana’s health sector.

Our observations in Ghana should be compared with responses to donor proliferation in other contexts. Future research should also consider how the composition of health-sector aid – e.g. the share directed at HIV and other disease-specific programmes – may influence responses to donor proliferation.
